# 

**Published:** 1804-10-01

**Authors:** 


### To CORRESPONDENTS.

The unusual influx of original matter, has obliged us to postpone our Analysis of Books, as well as several valuable Communications.

